# Body Influences on Social Cognition Through Interoception

**DOI:** 10.3389/fpsyg.2019.02066

**Published:** 2019-09-10

**Authors:** Qiyang Gao, Xianjie Ping, Wei Chen

**Affiliations:** ^1^Department of Psychology, Shaoxing University, Shaoxing, China; ^2^Center for Brain, Mind and Education, Shaoxing University, Shaoxing, China; ^3^Interdisciplinary Center for Philosophy and Cognitive Sciences, Renmin University of China, Beijing, China

**Keywords:** interoception, social cognition, visceroception, proprioception, body

## Introduction

From the dichotomy of body and mind to embodied cognition, the body as a material entity has become increasingly important in the cognitive development of individuals. Embodied cognition strengthens the current view that the body plays an essential role in cognition. For example, regarding the influence of body anatomy on cognition, if we replace human eyes with a bat's echolocation system, we will perceive the world in a completely different way (Goldman and de Vignemont, 2009). The impact of bodily activity on cognition is illustrated by better word memory and mathematical problem solving resulting from the use of hand gestures (Macedonia, 2014; Cook et al., 2017). The influence of bodily content and bodily format on cognition is shown when participants who reviewed resumes with heavy clipboards rather than light clipboards believed a candidate was more serious and had a better overall evaluation of that candidate (Ackerman et al., 2010). Regrettably, these studies focus on the causal effects of the body on cognition and ignore the influence of the internal state of the body on cognitive processing. Body ownership is a fundamental dimension of the bodily self which refers to the feeling that my body belongs to me, and the experience of body ownership is moderated by interindividual differences in the sensitivity to stimuli originating inside of the body, that is, by interoception (Schauder et al., 2015; Chen et al., 2017). It has been proposed that interoception also can moderate embodiments (Häfner, 2013). Interoception refers to the perception of internal body states; it is the intermediary between body and cognition. Several studies have demonstrated that interoception plays an important role in implicit memory processes and intuitive decision making (Dunn et al., 2010; Werner et al., 2010). Therefore, an “embodied sense of self” or “sense of embodiment” can become a new indicator of embodied cognition (Kilteni et al., 2012).

Social cognition is “a sub-topic of social psychology that focuses on how people process, store, and apply information about other people and social situations. It focuses on the role that cognitive processes play in social interactions” (Park et al., 2015). To date, an embodiment perspective has motivated novel insights about the understanding of others' minds (Wilson and Foglia, 2017), but how the body affects social cognition is still unclear. In this paper, we provide evidence that the body influences one's social cognition through interoception.

Interoception, as first proposed by Sherrington in 1906, originally only was referred to as visceroception (such as heart rate, breath, and hunger, etc.), and it did not include organs like the brain and skin (Tsakiris, 2017). It now refers to the ability to detect the feelings of internal bodily sensations, including proprioception (signals from the skin and musculoskeletal apparatus) and visceroception (Herbert and Pollatos, 2012; Schauder et al., 2015; Pollatos et al., 2016). Activation of interoceptive representations and meta-representations of bodily signals supporting interoceptive awareness are closely related to emotional experience and cognitive functions (Herbert and Pollatos, 2012). In this paper, we first explore interoception in general, review studies about proprioception and visceroception, and discuss the related brain regions. Thereafter, we summarize the relevant studies showing the association of social cognition with interoception in Table 1. Since the core components of social cognition are still the subject of some debate, we focus here on the traditionally associated processes: theory of mind (ToM), empathy, and imitation (Happé et al., 2017). Finally, we suggest that improving the sensitivity or accuracy of interoception can enhance one's ability to understand the mental state of others, thereby enabling better social communication.

**Table 1:** Relevant studies showing the association of social cognition with interoception.

**Study**	**Participants**	**Instruments/methods**	**Main findings**	**Attributies/neural correlates of social cognition**
Shah et al. (2017)	72 healthy adults	MASC, the heartbeat tracking task	Visceroception plays an important role in understanding emotion.	ToM
Fukushima et al. (2011)	21 healthy undergraduate students	EEG, ECG, the affective-judgment task, and the physical-judgment task	Visceroception may contribute to inferring the affective state of others.	Empathy
Grynberg and Pollatos (2015)	93 healthy undergraduate students	The Self-Assessment Manikin, the heartbeat perception task	Visceroception can shape both affective and cognitive empathy.	Empathy
Terasawa et al. (2014)	30 healthy undergraduate and graduate students	The heartbeat detection task and the emotional sensitivity task	Visceroception modulates the intensity of the emotional experience.	Empathy
Ernst et al. (2013)	18 healthy adults	fMRI, the empathy task, and the interoceptive task	The preceding visceral sensory awareness enhances neural activity in bilateral insula and various midline regions during empathy.	Empathy
Sedeño et al. (2014)	JM, a male patient with Depersonalization-Derealization Disorder and 10 age-matched healthy male	fMRI, the heartbeat detection task, and the empathy tasks	The impairments in the perception and integration of visceral information might lead to disruptions in affective empathic response.	Empathy
Haswell et al. (2009)	14 ASD children and 13 typically developing children	A motor task	The higher dependence on proprioception predicts the greater impairments in imitation.	Imitation
Izawa et al. (2012)	23 ASD children, 17 ADHD children, and 20 typically developing children	A motor task	Dependence on proprioception leads to impaired ability to imitate.	Imitation
Greenfield et al. (2015)	31 ASD children, 29 chronological age-matched children, and 29 verbal mental-age-matched children	A MIRAGE task	A greater reliance on proprioception would impact on sensitivity to social processes such as empathy and imitation.	Empathy and imitation
Tsakiris et al. (2006)	15 healthy adults	PET, the rubber hand illusion task	The proprioceptive behavioral data is positively correlated with activity in the right posterior insula and right frontal operculum.	Empathy/the anterior insula, the anterior cingulate cortex, and the midcingulate cortex.
Critchley et al. (2004)	17 healthy subjects	fMRI, the heartbeat detection task	The right anterior insula supports a representation of visceral responses.	Empathy/the anterior insula, the anterior cingulate cortex, and the midcingulate cortex.
Ronchi et al. (2015)	A patient with injured right insula and 16 age-matched healthy subjects	ECG, the heartbeat detection task	The insula plays a key role in interoceptive-cardiac processing.	Empathy/the anterior insula, the anterior cingulate cortex, and the midcingulate cortex.

## Visceroception and Social Cognition

Visceroception is the perception of internal body signals. These signals are mainly generated in four systems: cardiovascular, respiratory, gastrointestinal, and urogenital (mainly corresponding to the heart, lungs, stomach, and bladder) as well as other internal organs in the body torso. Among these, the cardiovascular system is the focus of research (Tsakiris, 2017), and perceived heartbeat is the most important experimental measure of interoception (Häfner, 2013; Melloni et al., 2013). The interoception in these cases essentially refers to visceroception.

Theory of Mind (ToM), an important aspect of social cognition, refers to the everyday ability to attribute independent mental states to oneself and others in order to predict and explain behavior (Premack and Woodruff, 1978; Shah et al., 2017). Shah et al. (2017) investigated whether visceroception can predict the mental states or only play an important role in understanding emotion. In the experiment, the participants focused their attention by silently counting their heartbeats during intervals of varying duration and then completed a questionnaire [the Movie for the Assessment of Social Cognition (MASC)] on ToM. The results showed that there was a positive correlation between interoceptive accuracy and overall MASC score. However, interoception is not necessary for the representation of mental states. It helps to accurately represent mental states in situations where the process is reliant on emotional or otherwise interoceptive information. That is, visceroception may be more relevant to the emotional aspects of social cognition.

Empathy—the understanding of the affective states of others (Fukushima et al., 2011)—is also an aspect of social cognition and plays a vital role in daily social activities. Fukushima et al. (2011) used the heartbeat-evoked potential (HEP) as a neural index of visceroception processing. Participants performed empathy tasks while electroencephalography (EEG) and electrocardiogram (ECG) measurements were recorded. The results showed that afferent feedback from visceral activity may contribute to inferring the affective state of others. Another study used a heartbeat perception task to prove that visceral sensitivity can shape both affective and cognitive empathy. Grynberg and Pollatos (2015) observed that participants with greater visceroception sensitivity overestimated the degree of pain (cognitive empathy), as well as arousal and feelings of compassion (affective empathy) when they saw pictures depicting other people in pain.

In neurophysiology, studies have shown that the anterior insula, the anterior cingulate cortex, and the midcingulate cortex are associated with empathy, and the insular cortex is also associated with the affective processing of self and others, and a wide range of social cognition processes (Bernhardt and Singer, 2012). The insula is also the central interoceptive hub in the brain (Craig and Craig, 2009). Ronchi et al. (2015) reported a single-case study showing that heartbeat awareness decreased after insular resection. In addition, Critchley et al. (2004) used functional magnetic resonance imaging (fMRI) and found that the activity of the insula, somatomotor cortex, and cingulate cortex was enhanced during the heartbeat perception task. Taken together, the overlap between the brain area related to visceroception and empathy further highlights visceroception as an important role for social cognition.

## Proprioception And Social Cognition

The term “proprioception” was introduced by Sherrington (1906) as the function of providing information about limb position and movement and, through the vestibular system, about balance and related whole-body properties (Gallagher, 2011).

Proprioception is closely related to the understanding of the intentions and beliefs of others. The account of the physician and philosopher Thomas Fuchs highlights the role of proprioception as a resource of understanding others. In his opinion, the proprioceptive skills of a physician can support the empathic understanding of the physician (Schmidsberger and Löffler-Stastka, 2018).

In addition, proprioception also has a regulating effect on imitation, an important aspect of social cognition. Proprioception normally promotes the development of social cognition, however patients with autism spectrum disorder (ASD) show the opposite situation. ASD children not only have defects in the development of social skills, but also have defects in the development of motor skills. In a study in which children learned to control a robotic arm to reach toward a target, Haswell et al. (2009) found that children with ASD placed a greater reliance on proprioception alone, and the internal models that they form place a stronger than normal association between the self-generated motor commands and proprioception, which indicates that sports, imitation, and social skills are compromised. As such, the higher dependence on proprioception, the more serious damage to social function and imitation. The same results have also been recognized by other researchers (Izawa et al., 2012).

Evidence indicates that social functioning deficits of children with ASD may be related to an over-reliance on proprioception or atypical visuo-tactile temporal binding (Cascio et al., 2012). It has been shown that ASD patients do have temporally extended visuo-tactile binding, which may result in reduced processing of temporal synchrony inputs over proprioception. This would likely lead to failures in appropriately binding information from related events, which would in turn impact sensitivity to social processes such as empathy and imitation (Greenfield et al., 2015).

Quattrocki and Friston (2014) gave another explanation for the abnormal behavior of ASD, suggesting that autism is caused by a dysfunctional oxytocin system in early life. Oxytocin has specific and targeted effects on the medial prefrontal cortex, the insula, the anterior cingulate, and the inferior frontal gyrus (Ninan, 2011; Riem et al., 2011). The distribution of oxytocin receptor in the brain supports the role of oxytocin in the transmission and modulation of interoceptive signals (prediction errors) in neuronal circuits. Oxytocin can inhibit the interoceptive signal and play an important role in the attenuation of it. The attenuation of interoceptive sensing may also be a necessary condition for the emergence of cognitive empathy and compassionate behavior. In addition, clinical studies have suggested that exogenous oxytocin improved empathy and the ability to infer the mindset of others (Bartz et al., 2010), demonstrating the important impact of interoception on social cognition.

## Conclusion

Interoception refers to the perception of internal body states, playing an important role in the mental representation of the self. Self-representation is indispensable for a wide range of social cognition processes. In this paper, we demonstrated that interoception can influence social cognition, that is, the body can influence the individual's social cognition through interoception.

Specifically, improving one's interoceptive sensitivity or interoceptive accuracy can improve one's social cognition. Studies have shown that a tendency to focus on one's own internal experiences, or to conduct experimental self-observation, such as looking at oneself in a mirror and gazing at a self-photograph and at self-relevant words, can improve the accuracy of heartbeat perception (Ainley et al., 2012). Combined with the viewpoint of this paper, the above method can improve one's interoceptive sensitivity and improve social cognition accordingly.

Children are developing individuals, and their social cognition has a high degree of plasticity (Frick et al., 2014). It is more convenient to improve children's social cognition through interoception, and it may achieve good practical results. Future research can start from this aspect, recruiting participants of the corresponding age stage to test the validity of this view.

## Author Contributions

All authors listed have made a substantial, direct and intellectual contribution to the work, and approved it for publication.

### Conflict of Interest Statement

The authors declare that the research was conducted in the absence of any commercial or financial relationships that could be construed as a potential conflict of interest.
